# EEG channel selection based on sequential backward floating search for motor imagery classification

**DOI:** 10.3389/fnins.2022.1045851

**Published:** 2022-10-21

**Authors:** Chao Tang, Tianyi Gao, Yuanhao Li, Badong Chen

**Affiliations:** ^1^Institute of Artificial Intelligence and Robotics, Xi'an Jiaotong University, Xi'an, China; ^2^Institute of Innovative Research, Tokyo Institute of Technology, Yokohama, Japan

**Keywords:** electroencephalogram (EEG), channel selection, sequential backward floating search (SBFS), motor imagery (MI), brain-computer interface (BCI)

## Abstract

Brain-computer interfaces (BCIs) based on motor imagery (MI) utilizing multi-channel electroencephalogram (EEG) data are commonly used to improve motor function of people with motor disabilities. EEG channel selection can enhance MI classification accuracy by selecting informative channels, accordingly reducing redundant information. The sequential backward floating search (SBFS) approach has been considered as one of the best feature selection methods. In this paper, SBFS is first implemented to select the optimal EEG channels in MI-BCI. Further, to reduce the time complexity of SBFS, the modified SBFS is proposed and applied to left and right hand MI tasks. In the modified SBFS, based on the map of EEG channels at the scalp, the symmetrical channels are selected as channel pairs and acceleration is thus realized by removing or adding multiple channels in each iteration. Extensive experiments were conducted on four public BCI datasets. Experimental results show that the SBFS achieves significantly higher classification accuracy (*p* < 0.001) than using all channels and conventional MI channels (i.e., C3, C4, and Cz). Moreover, the proposed method outperforms the state-of-the-art selection methods.

## 1. Introduction

Brain-computer interface (BCI) refers to a complete system that processes signals from human brain to control different communication devices (Gao et al., 2021). With the advantages of non-invasiveness, portability, low cost, and high temporal resolution, electroencephalogram (EEG) is widely used in BCI systems (Padfield et al., 2019). Potential signals that are commonly used in EEG-based BCI system mainly include P300 evoked potentials (Picton, 1992; Li et al., 2010), steady state visually evoked potentials (SSVEP) (Wang et al., 2008; Zhang et al., 2018), and event-related desynchronization/synchronization (ERD/ERS) (Pfurtscheller and Da Silva, 1999; Pfurtscheller and Neuper, 2006).

Compared with stimuli-based BCI, the potential signals for motor imagery (MI) (Ang and Guan, 2016; Yang et al., 2020) can be easier acquired without external stimulus. MI tasks can bring out cortical rhythm amplitude suppression (ERD) and enhancement (ERS) over primary sensorimotor areas (Taniguchi et al., 2000; Neuper et al., 2005). According to ERD/ERS phenomenon, the corresponding imagery category can be determined. Therefore, it is of great significance to select brain area with active neural activities as signal sources to improve the quality of EEG signals. Excessive channels not only deteriorate the portability of BCI system, but also increase the difficulty of signal analysis (Handiru and Prasad, 2016). Selecting appropriate EEG channels for different subjects can improve the performance of MI-based BCI system.

According to the prior knowledge of neurology, C3, C4, and Cz electrodes and their surrounding channels contain the most information related to MI, for which these specific channels are commonly selected. Although the experience-dependent artificial channel selection is easy for preparation and implementation, it could be not sufficient for each subject. The popular channel selection schemes (Alotaiby et al., 2015) for MI can be mainly divided as embedded techniques, filtering techniques (Baig et al., 2020), wrapper techniques, etc. Embedded techniques integrate the channel selection processes with the model training process, such as recursive channel estimation with the training of support vector machine (SVM) (Lal et al., 2004; Schröder et al., 2005). Filtering techniques are usually based on EEG signal statistics such as common spatial pattern (CSP) filter coefficients (Tam et al., 2011) and specific criteria such as mutual information (Ang et al., 2012). Wrapper techniques typically adopt wrapper approachs with complete (Kamrunnahar et al., 2009), random (Wei and Wang, 2011) or sequential (Qiu et al., 2016) search strategies for subset channel selection (Arvaneh et al., 2010). In addition, neural network genetic method (Yang et al., 2012) and bispectrum-based method (Jin et al., 2020) were investigated for EEG channel selection. Recently, neurophysiological approaches based on correlation (Jin et al., 2019) and Granger causality (Varsehi and Firoozabadi, 2021) have also been used in MI channel selection. However, the EEG channel selection methods of existing studies have either shown unsatisfactory performance or can only be used for specific types of data (Varsehi and Firoozabadi, 2021).

Sequential backward floating search (SBFS) is a well-known feature selection method which has been used to process various physiological signals (Tork et al., 2013; Karnaukh et al., 2018; Ahirwal, 2021) and to perform body state assessments (Dreißig et al., 2020). In this paper, SBFS is utilized in EEG channel selection for MI-based BCI. The main contributions of this paper are as follows:

1) To the best of our knowledge, this is the first time SBFS has been utilized for EEG channel selection.

2) The modified SBFS was proposed and applied to left and right hand MI tasks to reduce the time complexity of SBFS.

3) Extensive experiments were conducted on four datasets to confirm the effectiveness of the proposed method.

The remainder of this paper is detailed as follows. Section 2 describes the data used in this paper and the proposed methods. Section 3 presents the results. The discussion is provided in Section 4, and finally we conclude the paper in Section 5.

## 2. Materials and methods

### 2.1. Datasets

In this work, four common public datasets were used to evaluate the proposed methods. All EEG data were collected from the subjects' brain through acquisition equipments rather than artificially generated.

**Figure 1:**
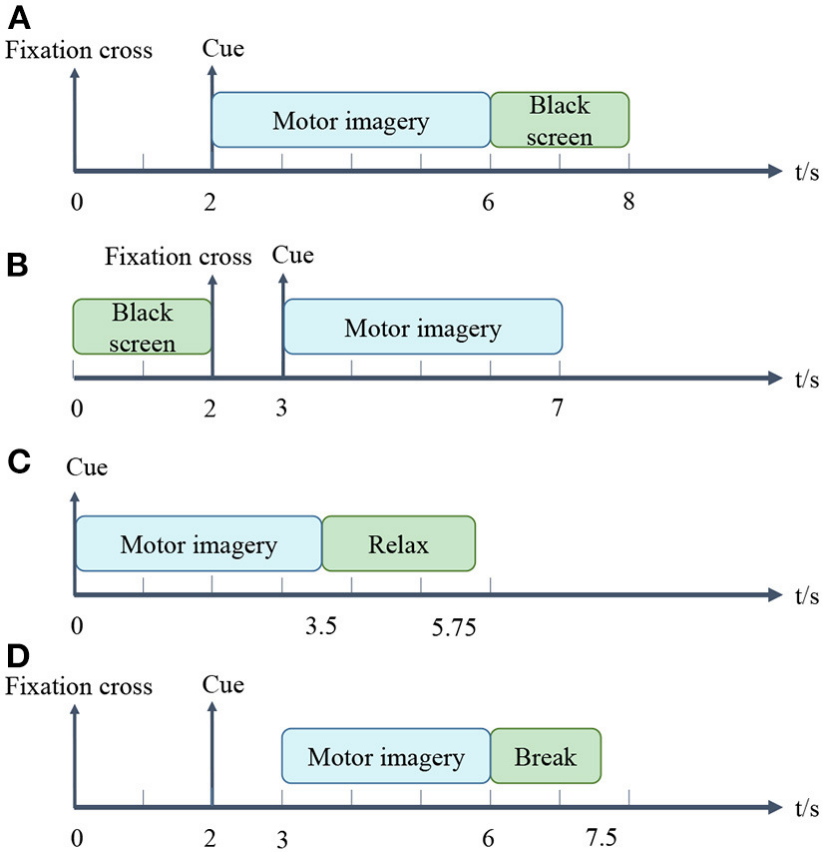
Time interval of one trial. **(A–D)** is for dataset 1), 2), 3) and 4), respectively.

*1) BCI Competition IV-dataset 1:* This dataset recorded 59 channels of EEG signals from 7 healthy subjects (Tangermann et al., 2012). We only used the data collected from subject a, b, f, and g, since the other data were artificially generated. Each subject participated in two classes (from the three classes left hand, right hand, and foot) of MI tasks. Each data included two runs, where each run contained 100 trials. In these two runs, arrows pointing left, right or down were displayed on the screen for visual cues. Cues were shown for a period of 4 s, during which the subjects were asked to perform the MI task. After and before the task, there were 2 s of blank and 2 s of display with a fixation cross presented in the center of the screen. Namely, the fixation cross was superimposed on the cues for 6 s. Each trial for the EEG data acquisition is illustrated in Figure 1A. The EEG signals were downsampled to 100 Hz.

*2) BCI Competition III-dataset IIIa:* The dataset was recorded from 3 subjects (k3, k6, and l1) in 60 channels with a sampling rate of 250 Hz (Blankertz et al., 2006). The subjects performed imagery left hand, right hand, foot or tongue movements according to a cue of random order. When a trial began, the first 2 s were quiet black-screen. Then an acoustic stimulus and a cross “+” were presented at *t* = 2 s. From *t* = 3 s an arrow pointing to left, right, up or down was shown for 1 s. In the meantime, the subjects imagined the movement corresponding to the arrow until *t* = 7 s. Each trial for the EEG data acquisition is shown in Figure 1B. The number of trials per class was 90 or 60 for different subjects. We only use the left and right hand MI trials in this study.

*3) BCI Competition III-dataset IVa:* The dataset was recorded from 5 healthy subjects (aa, al, av, aw, and ay) (Blankertz et al., 2006). The subjects performed one of the left hand, right hand and right foot MI within 3.5 s of the occurrence of the visual cues. Target cues were presented at random intervals (1.75–2.25 s), during which subjects could relax. Each trial for the EEG data acquisition is presented in Figure 1C. Each subject participated in 280 trials. The EEG signals were recorded with 118 channels and were downsampled at 100 Hz.

*4) BCI competition IV-dataset 2a:* The dataset recorded the EEG data of 9 subjects (A01–A09) who participated in the 4-class (left hand, right hand, both feet, and tongue) MI experiments (Tangermann et al., 2012). Raw data were collected at 22 channels and 250 Hz sampling rate. Each subject's data were recorded in 2 sessions, each session consisted of 6 runs, and each run contained 48 trials. i.e., each session was composed of 288 trials in 4 classes, and each class contained 72 trials. We only classified the trials of left hand and right hand in Session 1. The timeline for a trial is about 7.5 s, as detailed in Figure 1D. At the beginning (*t* = 0 s), a cross “+” appeared on the black screen. After 2 s (*t* = 2 s), an arrow pointing either to the left, right, down or up appeared and stayed on the screen for 1.25 s. The subjects performed the desired MI tasks until *t* = 6 s. After a short break, the screen went black again.

### 2.2. Data preprocessing

**Figure 2:**
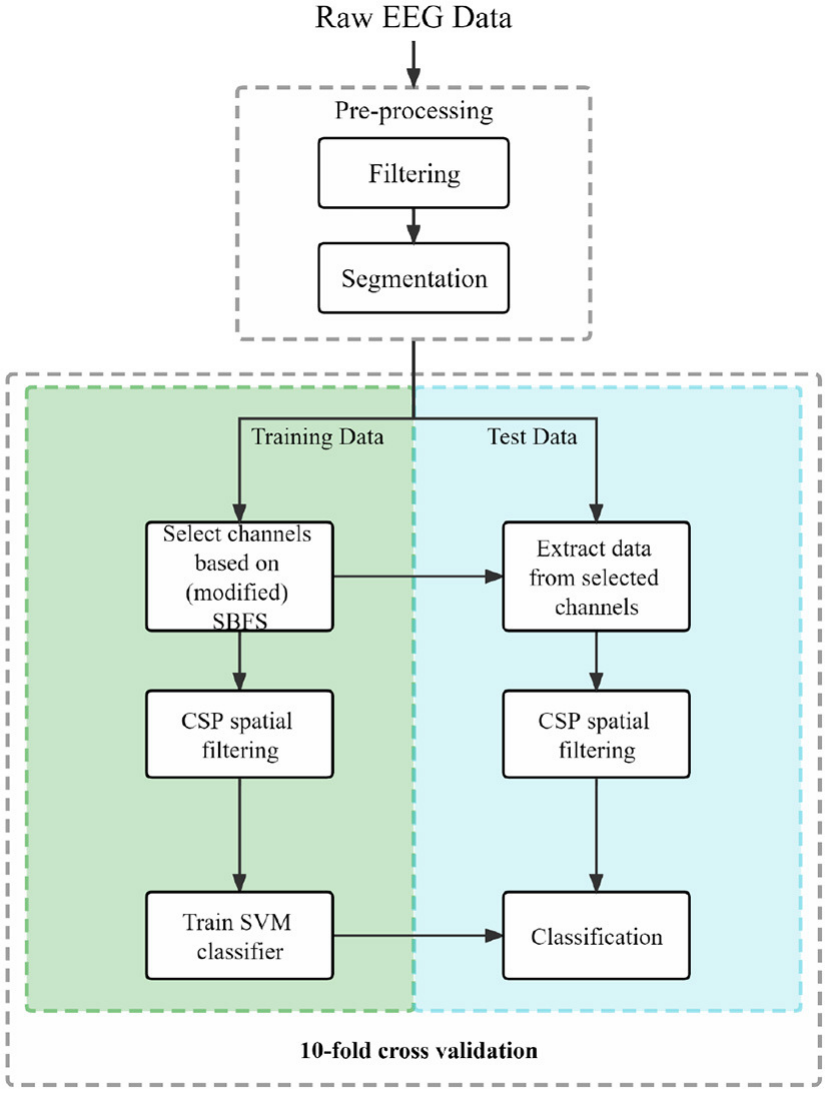
Block diagram of the proposed framework.

The acquired EEG data were refined in the preliminary analysis prior to channel selection, feature extraction, and classification. A portion of Figure 2 shows the preprocessing procedure.

*1) Filtering:* A third-order butterworth filter was applied to raw EEG data in the filtering part. The EEG data from each trial were filtered between 8 and 30 Hz.

*2) Segmentation:* The filtered EEG data were segmented by extracting data segments related to event types. MI events mainly consist of two intervals: MI and other states (rest or black screen). For dataset 1), 2), 3) and 4), we used fixed time windows of 2–6 s, 3–6 s, 0–3 s, and 3–6 s, respectively. More details exhibit in Figure 1.

### 2.3. Channel selection

#### 2.3.1. SBFS-based channel selection

The purpose of channel selection is to identify important channels and remove redundant and irrelevant channels. The SBFS starts with a complete set, which is based on a top-down approach (Pudil et al., 1994). We investigated the SBFS method for EEG channel selection in MI classification. In this study, *Y* denotes the entire channel set. *X*_*k*_ denotes the subset of channels containing *k* channels. *J*(*X*_*k*_) denotes the classification performance of a subset *X*_*k*_. The SBFS algorithm for channel selection is given in Algorithm 1.

**Algorithm 1:** EEG channel selection using SBFS.

**Input**: the set of all channels, *Y* = {*y*_1_, *y*_2_, ..., *y*_*d*_}
• The SBFS algorithm takes the entire channel as input. **Output**: *X*_*k*_ = {*x*_*j*_|*j* = 1, 2, ..., *k*; *x*_*j*_ ∈ *Y*}, where *k* = (0, 1, 2, ..., *d*)
• SBFS returns a subset of channels; the number of selected channels *k*, where *k* < *d*. **Initialization**: *X*_*k*_ = *Y, k* = *d*
• We initialize the algorithm with the given channel set such that *k* = *d*. **Step 1(Exclusion):**
$x^{-}=arg\;max\;J\left(X_{k}-x\right),where\;x\in X_{k}$
$X_{k-1}=X_{k}-x^{-}$
*k* = *k* − 1
Go to Step 2
• In step 1, we remove a channel *x*^−^ from the channel subset *X*_*k*_.
• *x*^−^ is the channel that maximizes our criterion function upon removal, that is, the channel which is associated with the optimal classification performance if it is removed from *X*_*k*_. **Step 2(Conditional Inclusion):**
$x^{+}=arg\;max\;J\left(X_{k}+x\right),where\;x\in Y-X_{k}$
*if J*(*X*_*k*_ + *x*) > *J*(*X*_*k*+1_):
$X_{k+1}=X_{k}+x^{+}$
*k* = *k* + 1
Go to Step 1
• In step 2, we search for channels that would improve the classification performance when added back to the channel subset. If such channels exist, we add the channel *x*^+^ that maximizes the performance improvement. If *k* = *n* or an improvement cannot be made (i.e., such channel *x*^+^ cannot be found), go back to step 1; else, repeat the current step. **Termination:** *k* = 2
• The channel subset of size *k* contains the desired number of channels 2.

The advantage of applying the update strategy to SBFS in EEG channel selection is the possibility to increase the value of optimal accuracy or decrease the number of channels of optimal accuracy. It is described as follows: the SBFS algorithm pursues the maximum accuracy under the current number of channels, and the intermediate (Inclusion) process of the later channel selection may result in the increase of the accuracy of the previous number of channels. Our update strategy is to replace with the maximum accuracy each time.

#### 2.3.2. Reducing time complexity: Modified SBFS

**Figure 3:**
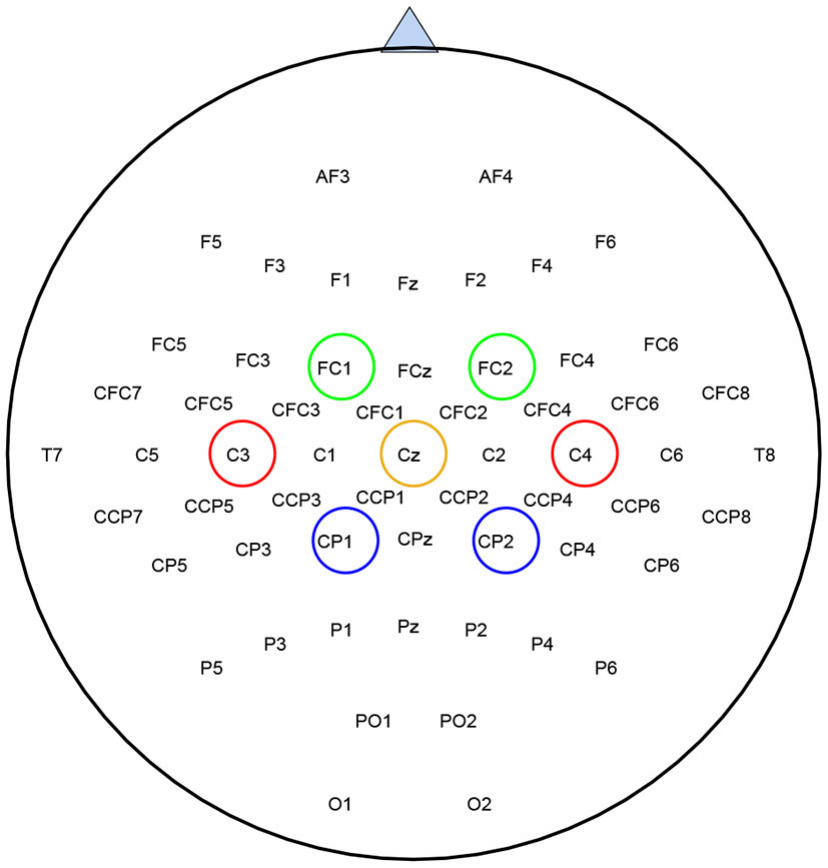
Location of EEG electrodes used for data acquisition, taking BCI competition IV-dataset 1 as an example. Channels of the same color are treated as a channel pair for selection.

**Figure 4:**
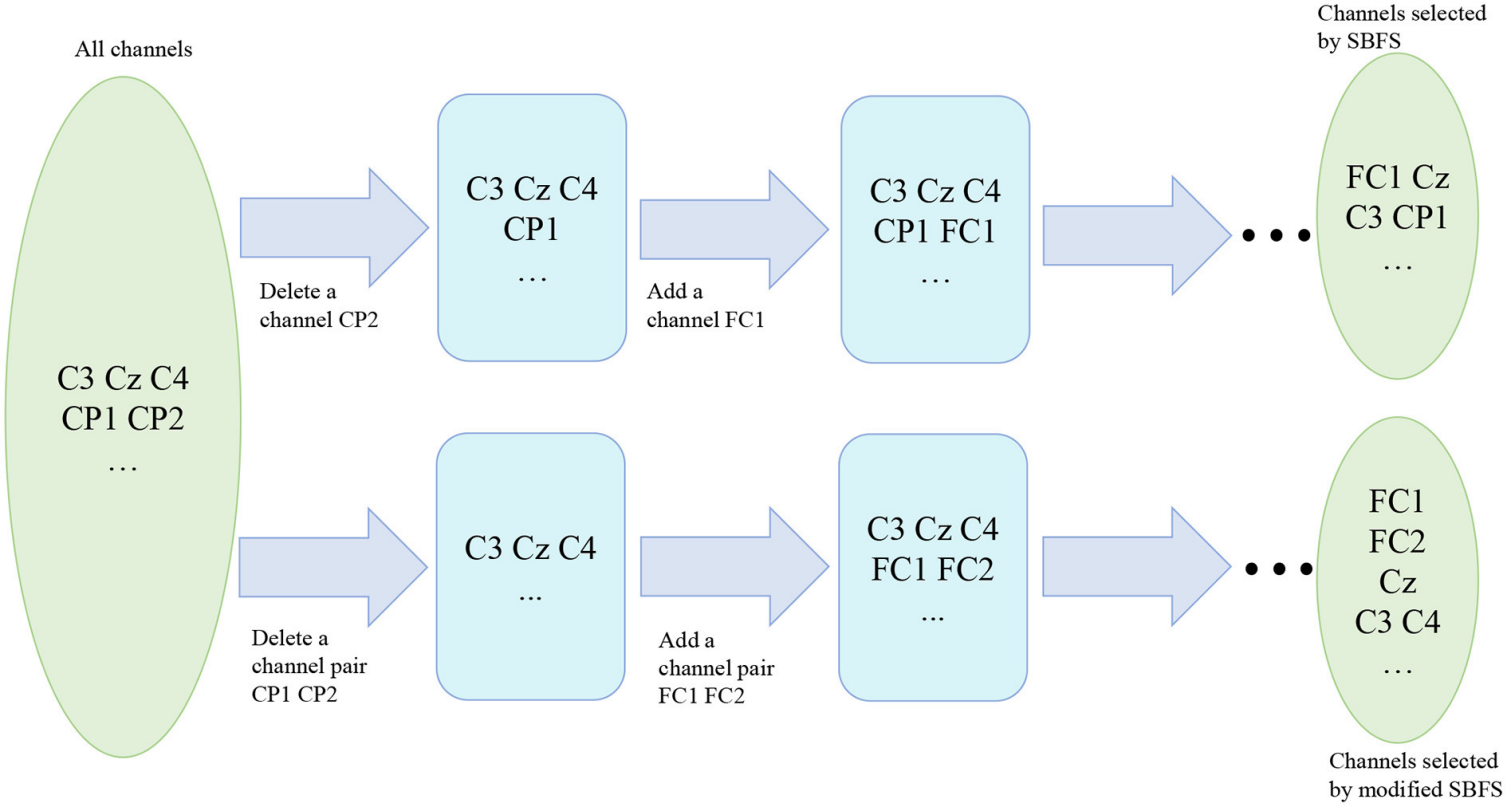
Search chart of the SBFS and the modified SBFS.

Since SBFS is a search method, it makes sense to speed up the search process without compromising accuracy. Considering that mu (8–13 Hz bands) and beta (14–30 Hz bands) ERD/ERS phenomenon are elicited during imagined hand movements (Ramoser et al., 2000), depending on the location of the channels in the cerebral cortex, left-right symmetrical channels can be treated as a channel pair. As is shown in Figure 3, channels of the same color are considered as a channel pair. For example, red C3 and C4, blue CP1 and CP2, and green FC1 and FC2 are channel pairs, respectively. Both of them are left-right symmetrical with respect to the straight lines of CZ and CPZ. Thus, the whole set contains fewer channel pairs and the modified SBFS can remove or add multiple channels at a time. The main differences between SBFS and modified SBFS methods are shown in Figure 4. One can observe that the modified SBFS contains far fewer channel pairs than before. The time spent on searching process can be greatly reduced.

### 2.4. Feature extraction

CSP (Ramoser et al., 2000) is an efficient feature extraction algorithm for binary classification tasks, which has been extensively used in MI-based BCI (Dong et al., 2017; Chen B. et al., 2018). It finds a spatial filter to maximize the differences in variance between two classes of multi-channel EEG data. Let **C**_*a*_ and **C**_*b*_ be the normalized covariance matrices of the two classes of EEG signals which are averaged over trials. The composite spatial covariance matrix is **C**_*c*_ = **C**_*a*_ + **C**_*b*_. Decomposing **C**_*c*_, we can obtain ${\text{C}}_{c}=\text{U}\lambda {\text{U}}^{\text{T}}$, where λ is the eigenvalue and **U** is the eigenvector. And the whitening transformation is


(1) $$\begin{array}{l} \text{P}=\sqrt{\lambda^{-1}}{\text{U}}^{\text{T}} \end{array}$$

Then the covariance matrices **C**_*a*_ and **C**_*b*_ can be transformed into:


(2) $$\begin{array}{l} {\text{S}}_{a}=\text{P}{\text{C}}_{a}{\text{P}}^{\text{T}} \end{array}$$

(3) $$\begin{array}{l} {\text{S}}_{b}=\text{P}{\text{C}}_{b}{\text{P}}^{\text{T}} \end{array}$$

It can be seen that **S**_*a*_ and **S**_*b*_ have the same eigenvector. Then we have **S**_*a*_ + **S**_*b*_ = **I**. Any orthonormal matrix **V** satisfies the following


(4) $$\begin{array}{l} {\text{V}}^{\text{T}}\left({\text{S}}_{a}+{\text{S}}_{b}\right)\text{V}=\text{I} \end{array}$$

Using the orthonormal matrix **V**, **S**_*a*_ and **S**_*b*_ can be decomposed as follows:


(5) $$\begin{array}{l} {\text{S}}_{a}=\text{V}\Lambda_{a}{\text{V}}^{\text{T}} \end{array}$$

(6) $$\begin{array}{l} {\text{S}}_{b}=\text{V}\Lambda_{b}{\text{V}}^{\text{T}} \end{array}$$

and Λ_*a*_ + Λ_*b*_ = **I**. The projection matrix is


(7) $$\begin{array}{l} \text{W}={\text{P}}^{\text{T}}\text{V} \end{array}$$

**W** is called the CSP weight matrix. The optimal features can be obtained in the least square case. Finally, the vector of the features is expressed as:


(8) $$\begin{array}{l} f=log\left(var\left(\text{W}\text{x}\left(t\right)\right)\right) \end{array}$$

where, **x**(*t*) is EEG data.

### 2.5. Classification

SVM theory was proposed by Vapnik (1999). The core idea of SVM is to separate the data from the two classes by finding a hyperplane with the largest possible margin. As one of the most commonly used BCI-based MI classifiers, SVM (Subasi and Gursoy, 2010; Qin et al., 2019) can effectively solve the classification problem of two classes of EEG data. In this study, we used an SVM with a radial basis function kernel to classify MI tasks after feature extraction. The separation of training and test data is realized by using 10-fold cross validation in the classification part.

### 2.6. Framework overview

Firstly, the raw MI EEG data of each subject were preprocessed. The SBFS and the modified SBFS method were applied to the training data to obtain the selected channels. The CSP spatial domain filter was applied for training data to acquire weight matrices. Finally, the SVM classifier was trained and the classification performance with 10-fold cross validation was obtained. The block diagram of our proposed framework is shown in Figure 2.

## 3. Results

### 3.1. Classification accuracy and significance

The classification accuracies of all subjects from datasets 1), 2), 3) using different methods are shown in Table 1. The classification accuracies of 14 subjects which participated left and right hand MI using different methods are shown in Table 2. The optimal classification accuracy for each subject and mean are in bold. The last row of Tables 1, 2 gives the paired *t*-test results of the SBFS or the modified SBFS with the current column method. The C3C4Cz method indicates that only EEG data from these 3 channels are used in the classification of MI. From Table 1, for each single subject, the highest classification accuracy was obtained with SBFS. In particular, subjects aw and ay achieved 100% classification accuracy. Compared with all channels, the average performance improvement of the SBFS method in datasets 1), 2), 3) was 18.8, 31.1, and 27.9%, respectively. Meanwhile, the SBFS method improved by 18.3, 31.3, and 29.7%, respectively, compared with the C3C4Cz method. From Table 2, the average accuracy of the SBFS and the modified SBFS is improved by 21.4 and 20.4%, respectively. It is shown that the accuracy of the SBFS is significantly better than all channels and conventional MI channels (*p* < 0.0001). There is no significant difference (*p* = 0.1522) between the SBFS and the modified SBFS.

**Table 1:** Comparison of classification accuracy (%) with different methods on datasets 1), 2), 3).

**Subject**	**Methods**		
	**All channels**	**C3C4Cz**	**SBFS**
a	52.0	43.5	**74.5**
b	44.0	51.0	**64.5**
f	49.0	46.5	**63.5**
g	50.0	63.0	**68.0**
Mean ± std	48.8 ± 3.4	49.3 ± 5.5	**67.6** ± 5.0
k3	45.6	53.3	**78.9**
k6	46.7	41.7	**76.7**
l1	50.0	46.7	**80.0**
Mean ± std	47.4 ± 2.3	47.2 ± 5.8	**78.5** ± 1.7
aa	62.1	58.8	**84.4**
al	62.3	74.1	**95.0**
av	47.5	60.0	**75.0**
aw	75.6	68.0	**100**
ay	67.5	45.0	**100**
Mean ± std	63.0 ± 10.3	61.2 ± 11.0	**90.9** ± 10.9
*P*-value	<0.0001	<0.0001	−

“−” Denotes the missing values. The optimal classification accuracies in each row are in bold.

**Table 2:** Comparison of classification accuracy (%) with different methods on 14 subjects which participate in left and right hand MI tasks.

**Subject**	**Methods**		
	**All channels**	**SBFS**	**Modified SBFS**
b	44.0	**64.5**	62.5
g	50.0	68.0	**69.0**
k3	45.6	**78.9**	74.4
k6	46.7	**76.7**	71.7
l1	50.0	80.0	**81.7**
A01	45.7	**70.7**	67.9
A02	51.4	**66.4**	65.7
A03	55.0	77.9	**79.3**
A04	46.4	62.9	**63.6**
A05	51.4	65.0	**67.9**
A06	49.3	**63.6**	61.4
A07	57.9	**69.3**	65.6
A08	60.7	**87.1**	**87.1**
A09	48.6	**71.4**	70.7
Mean ± std	50.2 ± 4.8	**71.6** ± 7.4	70.6 ± 7.6
*P*-value	<0.0001	0.1522	−

“−” Denotes the missing values. The optimal classification accuracies in each row are in bold.

### 3.2. Number of selected channels

The number of selected channels with optimal classification accuracy is shown in Table 3. From Table 3, the number of channels selected by the SBFS shows a substantial decrease compared to all channels. In terms of averages, this is specifically shown as 23 vs. 59, 12 vs. 60, 25 vs. 118, and 10 vs. 22. Overall, the number of selected channels ranges from one-fifth to one-half of the total number of usable channels. The number of channels selected by the modified SBFS method is similar to that of the SBFS.

**Table 3:** Comparison of the number of channels selected at the highest accuracy.

**Subject**	**Methods**		
	**All channels**	**SBFS**	**Modified SBFS**
a	59	10	−
b	59	45	14
f	59	4	−
g	59	33	17
Average	59	23	−
k3	60	10	14
k6	60	19	20
l1	60	6	20
Average	60	12^*^	18
aa	118	40	−
al	118	31	−
av	118	37	−
aw	118	6	−
ay	118	13	−
Average	118	25^*^	−
A01	22	7	9
A02	22	4	8
A03	22	13	17
A04	22	3	14
A05	22	7	13
A06	22	14	6
A07	22	19	19
A08	22	8	10
A09	22	15	9
Average	22	10	12^*^

“*” Denotes that the original number is not an integer.

“−” Denotes the missing values.

### 3.3. Computation time

**Figure 5:**
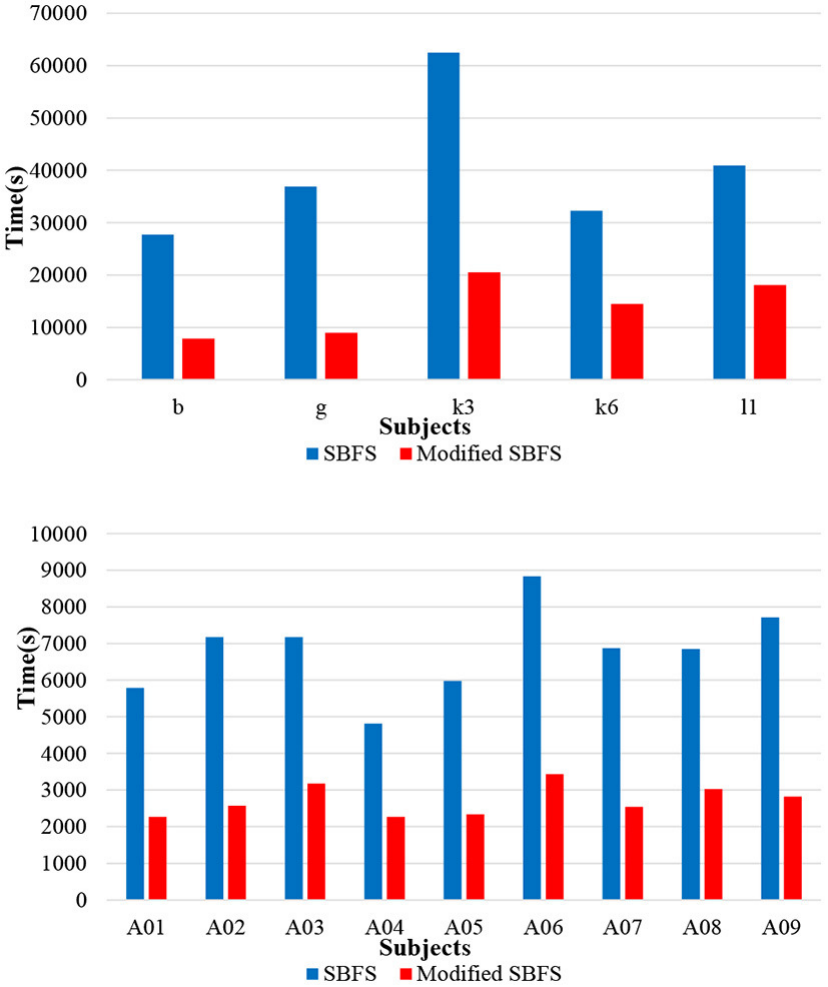
Computation time comparisons between SBFS and modified SBFS.

In order to compare the computation time between the SBFS method and the modified SBFS method, the results of 14 subjects who participated in a left and right hand MI task from datasets 1), 2), 4) were used. The two algorithms were implemented and tested using MATLAB 2019^1^ configured on Windows 10 professional operating system and the experiments were performed on an Intel (R) Core (TM) i5-8265U CPU @ 1.60GHz, 8.00 GB RAM computer. As can be seen from Figure 5, the modified SBFS method for channel selection is faster than the SBFS method. It is precisely because more than one channel were added or deleted each time that the number of iterations was reduced, which greatly saved the time.

The computation time of the method is affected by several factors, such as the number of all channels, the number of trials, the length of trials, software and hardware configuration etc. For example, using a parallel for loop (parfor) in Matlab to speed up the algorithm, the iterations of the parfor loop can run in parallel on multiple cores of the target hardware (our computer has 4 cores), and speed of the algorithm obtained by testing is shown to be about 4 times faster. During the initial sessions (also called the training sessions) in BCI experiments, the desired parameters are adjusted offline according to the signals collected from different subjects. Thus, the running speed of the channel selection part is not a concern, even for the SBFS method. In short, users have the flexibility to choose the SBFS or the modified SBFS methods according to specific practical situations.

### 3.4. Comparison with other selection methods

We compared SBFS-based EEG channel selection method with other algorithms in this field. For fairness of comparison, the data preprocessing, feature extraction and classifier were used identically.

CSP-rank (Tam et al., 2011) is a channel selection method in MI-based BCI using CSP. The method is based on the sorting of CSP filters. To be specific, we first rank the absolute values of the filter coefficients in each filter respectively, and then take the electrodes with the next largest coefficients from the two spatial filters in turn.

Improved sequential floating forward selection (ISFFS) (Qiu et al., 2016) combines the practical distribution of channels and an intelligent selection algorithm to select EEG channels.

Correlation-based channel selection (CCS) (Jin et al., 2019) assumes that there is a high correlation between task-related channels, then the relevant channels are selected.

Tables 4, 5 presents the classification accuracy and the number of channels at optimal accuracy for different methods, respectively. The optimal classification accuracy for each subject and mean are in bold. The SBFS method achieved the best classification accuracy for both single subjects and mean values. The SBFS and the ISFFS methods are similar in the number of selected channels, less than the CSP-rank and the CCS method.

**Table 4:** Comparison of classification accuracy (%) with different channel selection methods on BCI competition IV-dataset 1.

**Subject**	**Methods**			
	**CSP-rank**	**ISFFS**	**CCS**	**SBFS**
a	62.0	64.5	57.5	**74.5**
b	57.5	61.5	58.0	**64.5**
f	53.5	63.5	55.0	**63.5**
g	61.5	63.5	63.0	**68.0**
Mean	58.6	63.3	58.4	**67.6**

The optimal classification accuracies in each row are in bold.

**Table 5:** Comparison of the number of selected channels with different channel selection methods on BCI competition IV-dataset 1.

**Subject**	**Methods**			
	**CSP-rank**	**ISFFS**	**CCS**	**SBFS**
a	53	41	42	10
b	27	11	21	45
f	57	8	38	4
g	7	24	25	33
Average	36	21	32^*^	23

“*” Denotes that the original number is not an integer.

## 4. Discussion

### 4.1. Maps of the selected channels

**Figure 6:**
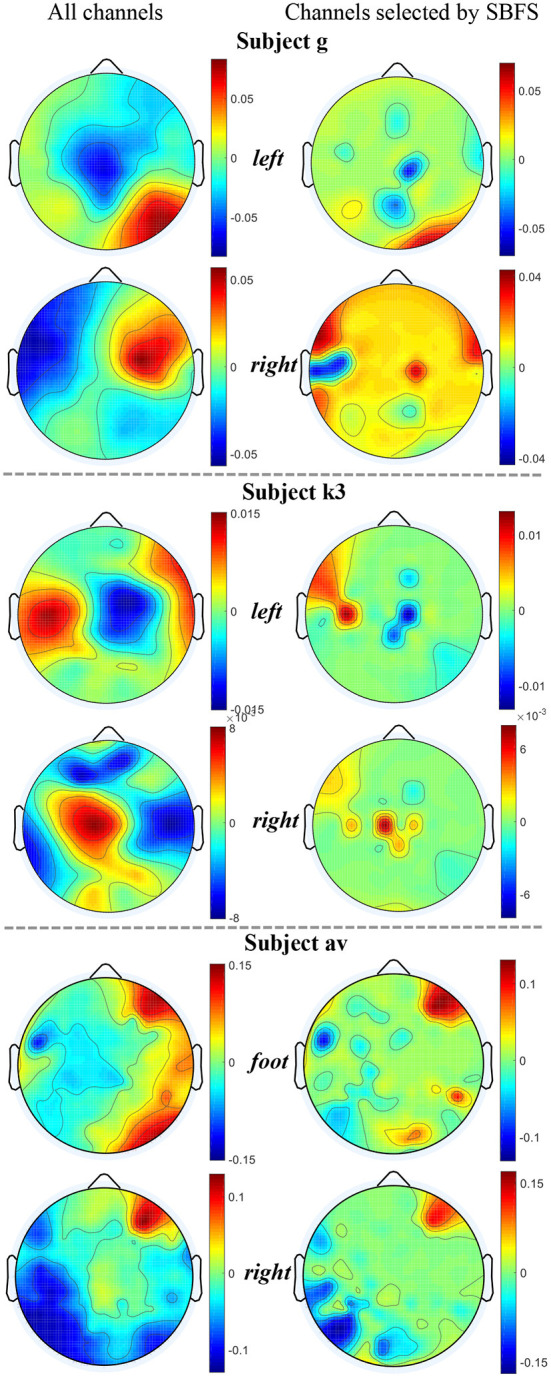
Topographic maps under two channel settings of subjects g, k3 and av from datasets 1), 2), 3). For each topographic map, the mean value of all trials in the training data is taken. The value of the unselected channels in the second column maps is set to 0.

**Figure 7:**
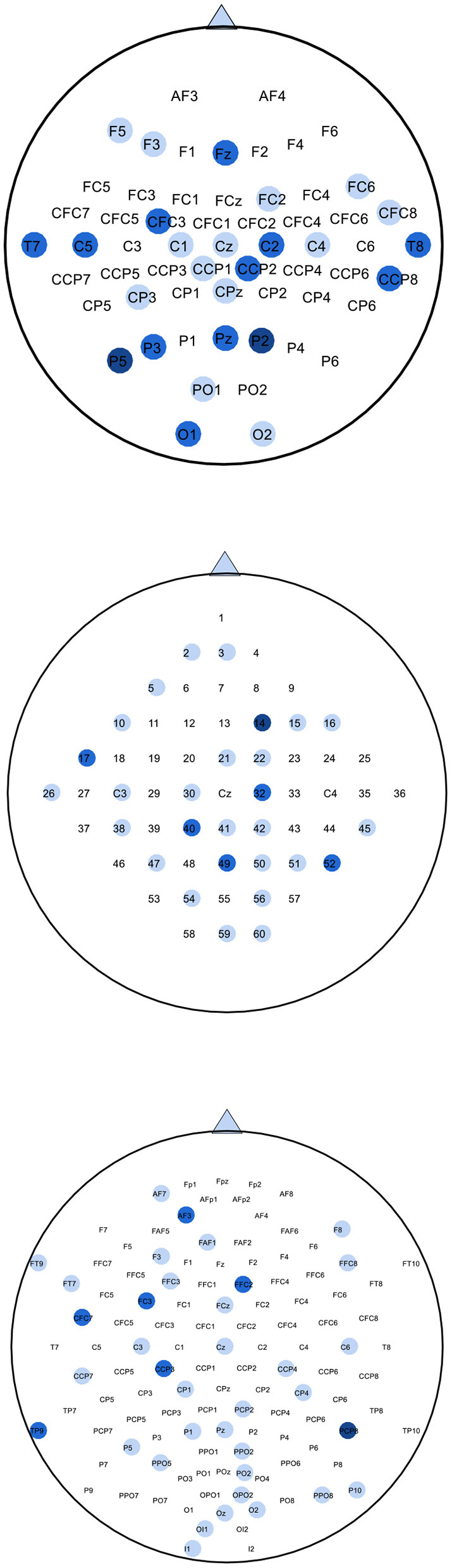
Maps of selected channels by SBFS for datasets 1), 2), 3). The blue circles represent selected channels. Darker colors indicate more selections. For dataset 1), channels selected more than twice from four subjects are colored in the top map. For dataset 2), channels selected more than once from three subjects are colored in the middle map. For dataset 3), channels selected more than twice from five subjects are colored in the bottom map.

We used MATLAB 2019 (see text footnote 1) with the EEGLAB toolbox (Delorme and Makeig, 2004) to plot topographic maps of subjects g, k3, and av from each of the datasets 1), 2), 3), as shown in Figure 6. The map of the channels selected by SBFS is shown in Figure 7.

The location of channels was compared with topographic maps. On the whole, the channels selected by SBFS were consistent with the corresponding ERD phenomena for all channels. Channel C3, C4, and Cz or their surrounding channels located in the motor area of the brain were selected multiple times. For dataset 1), Channel C4 and Cz were selected twice. Also, some channels (C2, C5, CFC3, and CCP2) around C3 and C4 were selected multiple times. Figure 6 shows that subject g in the right hand MI task, the ERD phenomena occurred mainly in the left cerebral cortex. For dataset 2), Channel C3 was selected. Channel 32 and 40, located around channel Cz, were also selected twice. In Figure 6, the ERD phenomena of subject k3 mainly occurred in the right cerebral cortex during left hand MI task. For dataset 3), all 5 subjects performed foot and right hand MI tasks. Channel C3 and Cz were selected twice. They were surrounded by channel CCP3 and CCP4 which were also selected multiple times. As shown in Figure 6, the ERD phenomena appears in the left cerebral cortex when subject av was performing a right hand MI task.

The SBFS method selected channels successively from bottom (i.e., serial number is larger) to top (i.e., serial number is smaller). This may lead to channels which are not related to MI being selected as well. The irregular channel positions resulted from an evaluation criterion by using cross-validation accuracy method. Many selected channels were located in the posterior part of the cerebral cortex. In the case of equal classification accuracy, the channels with larger ordinal numbers, i.e., the backward position, were retained, and the channels with smaller ordinal numbers, i.e., the forward position, were removed.

**Figure 8:**
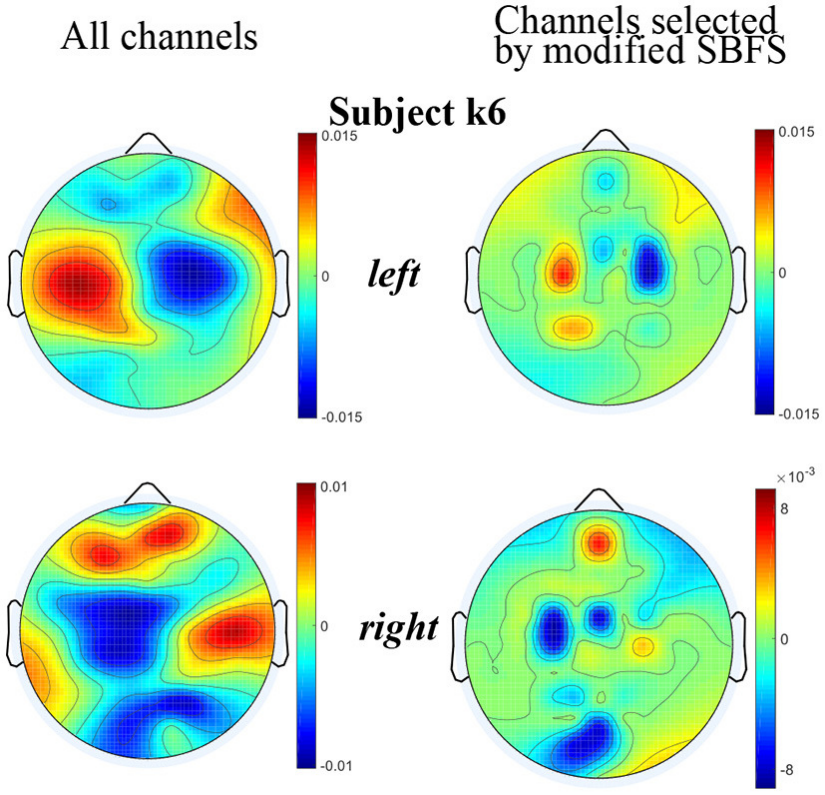
Topographic maps under two channel settings of subjects k6. For each topographic map, the mean value of all trials in the training data is taken. The value of the unselected channels in the maps is set to 0.

The topographic maps plotted using the EEGLAB toolbox under all channels and channels selected by the modified SBFS for subject k6 are shown in Figure 8, respectively. From Figure 8, it can also be seen that the topographic map of the channels selected by the modified SBFS was basically consistent with that of all channels. Specifically, clear ERD phenomenon can be observed in the left hand and right hand topographic maps under channels selected by modified SBFS.

### 4.2. Parameter sensitivity analysis

**Figure 9:**
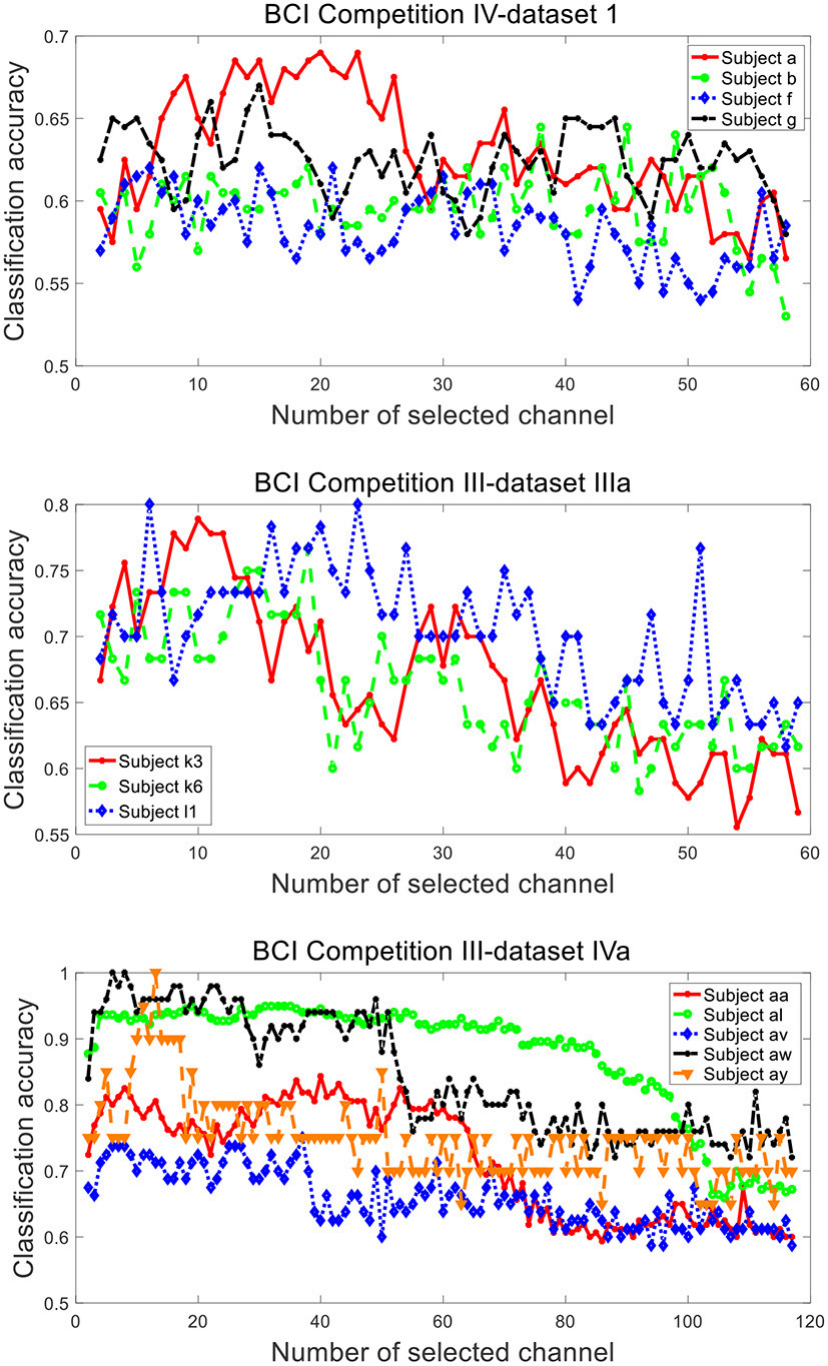
Classification accuracy of subjects from datasets 1), 2), 3) under different number of selected channels by SBFS.

Figure 9 plots the variation of classification accuracy with the number of selected channels by SBFS. With the increase of the number of selected channels, the overall trend of classification accuracy increases, then decreases. This may be due to the fact that there are few channels containing available information at the beginning and the initial accuracy is low. As the number of channels increases, the useful information keeps increasing and the accuracy is improved. With the sustainable increase of the number of channels, the number of redundant information channels increases as well, leading to the decrease of accuracy. Specifically, the change in accuracy with the number of channels is different for each subject. For example, the classification accuracies of subjects a, b, k6 and av have a slight decrease initially.

### 4.3. Future works

The SBFS method obviously outperforms other competing channel selection algorithms. In the following study, the proposed framework will be tested on datasets containing more subjects, such as the Physionet dataset (Goldberger et al., 2000), to strengthen sufficient persuasiveness. In addition, the SBFS can be combined with other features to further improve the classification performance in MI-based BCI. For example, for small sample data, it might be combined with regularized CSP feature (Lu et al., 2010).

In this paper, we studied the SBFS method for channel selection of MI tasks. The idea of a feature selection algorithm combined with task-related effective features can be extended to other tasks, e.g., for the SSVEP task, a combination of the SBFS and canonical correlation analysis (Lin et al., 2006) can be used, and for the rapid serial visual presentation task (Xu et al., 2021), a combination of the SBFS and hierarchical discriminant component analysis (Parra et al., 2007) can be used. Moreover, the proposed symmetrical strategy which is with respect to the optimization time can also be extended to other tasks, such as EEG-based emotion recognition (Chen T. et al., 2018; Gao et al., 2020; Tang et al., 2022).

Note that, ERD and ERS phenomenon are found not only in EEG but also in magnetoencephalography (MEG) recordings. As another non-invasive physiological signal, MEG-based BCI often involves more sensors. The existing MEG instrument based on superconducting quantum interference device technology is typically composed of 275 (gradiometer) or 306 (204 gradiometer and 120 magnetometer) sensors. Although a large number of sensors can provide higher spatial-temporal resolution for evaluating brain activity patterns, not all sensors can significantly improve classification accuracy. In addition, a larger number of channels implies a greater computation complexity. Recently, Roy et al. (2019, 2020) assessd the effect of channel selection using intelligent algorithms on MEG decoding of MI for the first time. Therefore, the application of the proposed method to MEG data can be explored in the future.

## 5. Conclusion

In this paper, the SBFS method is first applied to EEG channel selection, combining CSP features and an SVM classifier to form a new decoding framework. In order to reduce the time complexity of SBFS, the modified SBFS method is proposed, in which symmetrical channel pairs are removed or added in each iteration depending on the location of EEG channels at the scalp. Experimental results show that the proposed method can significantly improve the classification accuracy while reducing the number of EEG channels. The study provides a new approach to improve the reliability of future BCI systems.

## Data availability statement

The original contributions presented in the study are included in the article/supplementary material, further inquiries can be directed to the corresponding author/s.

## Author contributions

CT: data collection, methodology, and writing—original draft. TG: data collation and analysis. YL: writing—reviewing. BC: reviewing and supervision. All authors contributed to the article and approved the submitted version.

## Funding

This work was supported by the National Natural Science Foundation of China with Grant Nos. (U21A20485 and 61976175).

## Conflict of interest

The authors declare that the research was conducted in the absence of any commercial or financial relationships that could be construed as a potential conflict of interest.

## Publisher's note

All claims expressed in this article are solely those of the authors and do not necessarily represent those of their affiliated organizations, or those of the publisher, the editors and the reviewers. Any product that may be evaluated in this article, or claim that may be made by its manufacturer, is not guaranteed or endorsed by the publisher.

## Footnotes

^1^MATLAB is a commercial mathematical software from MathWorks, Inc. in the U.S. 2019a is the version number.
